# Prediction of therapy response in bone-predominant metastatic breast cancer: comparison of [^18^F] fluorodeoxyglucose and [^18^F]-fluoride PET/CT with whole-body MRI with diffusion-weighted imaging

**DOI:** 10.1007/s00259-018-4223-9

**Published:** 2018-12-01

**Authors:** Gurdip K. Azad, Benjamin P. Taylor, Adrian Green, Ines Sandri, Angela Swampillai, Mark Harries, Hartmut Kristeleit, Janine Mansi, Vicky Goh, Gary J. R. Cook

**Affiliations:** 1 Cancer Imaging Department, School of Biomedical Engineering and Imaging Sciences, King’s College London, London, UK; 2 Department of Oncology, Guys and St Thomas’ Hospital NHS Foundation Trust, London, UK; 3 King’s College London & Guy’s and St Thomas’ PET Centre, St Thomas’ Hospital, London, UK

**Keywords:** Whole-body MRI, Diffusion-weighted MRI, [^18^F]-fluorodeoxyglucose, [^18^F]-sodium fluoride, Positron emission tomography/computed tomography, Bone metastases

## Abstract

**Purpose:**

To compare [^18^F]-fluorodeoxyglucose (FDG) and [^18^F]-sodium fluoride (NaF) positron emission tomography/computed tomography (PET/CT) with whole-body magnetic resonance with diffusion-weighted imaging (WB-MRI), for endocrine therapy response prediction at 8 weeks in bone-predominant metastatic breast cancer.

**Patients and methods:**

Thirty-one patients scheduled for endocrine therapy had up to five bone metastases measured [FDG, NaF PET/CT: maximum standardized uptake value (SUV_max_); WB-MRI: median apparent diffusion coefficient (ADC_med_)] at baseline and 8 weeks. To detect the flare phenomenon, a 12-week NaF PET/CT was also performed if 8-week SUV_max_ increased. A 25% parameter change differentiated imaging progressive disease (PD) from non-PD and was compared to a 24-week clinical reference standard and progression-free survival (PFS).

**Results:**

Twenty-two patients (median age, 58.6 years, range, 40–79 years) completing baseline and 8-week imaging were included in the final analysis.

Per-patient % change in NaF SUV_max_ predicted 24-week clinical PD with sensitivity, specificity and accuracy of 60, 73.3, and 70%, respectively. For FDG SUV_max_ the results were 0, 100, and 76.2% and for ADC_med_, 0, 100 and 72.2%, respectively.

PFS < 24 weeks was associated with % change in SUV_max_ (NaF: 41.7 vs. 0.7%, *p* = 0.039; FDG: − 4.8 vs. − 28.6%, *p* = 0.005) but not ADC_med_ (− 0.5 vs. 10.1%, *p* = 0.098). Interlesional response heterogeneity occurred in all modalities and NaF flare occurred in seven patients.

**Conclusions:**

FDG PET/CT and WB-MRI best predicted clinical non-PD and both FDG and NaF PET/CT predicted PFS < 24 weeks. Lesional response heterogeneity occurs with all modalities and flare is common with NaF PET/CT.

## Introduction

Bone metastases in patients with advanced breast cancer are common, occurring in at least 70% of patients with advanced disease, and cause significant morbidity [1]. Patients with breast cancer and bone metastases have a relatively long survival compared to other cancers and coupled with the associated morbidity, there are significant implications for healthcare costs [2, 3]. Despite improved therapeutics, response rates are generally less than 50% and so accurate and timely treatment response-assessment methods are essential for optimal management [4]. However, it is recognized that there is an unmet clinical need for correct categorization of treatment response versus non-response in skeletal metastases at an early time point as conventional methods, e.g., RECIST 1.1 measurements on computed tomography (CT) or magnetic resonance imaging (MRI), usually classify skeletal metastases as non-measurable disease [5, 6]. Similarly, the isotope bone scan is considered to have poor sensitivity and specificity for detecting early response or non-response [7]. This means that without an objective early measure of non-response, patients with bone-predominant metastatic disease may continue with ineffective treatment longer than necessary, delaying therapeutic transition to second or third-line treatment and exposing them to unnecessary treatment-related side effects.

There is increasing evidence that functional imaging methods may be able to address this need with reported studies evaluating individual modalities. There is greatest supporting evidence for [^18^F]-fluorodeoxyglucose (FDG) positron emission tomography/computed tomography (PET/CT) measuring reduction in glucose metabolism in responding metastases [8–12]. There is relatively little evidence for alternative imaging methods, which nevertheless show promise in breast cancer or other cancers, including [^18^F]-sodium fluoride (NaF) PET/CT [13–17] and whole-body MRI including diffusion-weighted sequences (WB-MRI) [18–20]. NaF uptake is dependent on altered blood flow and mineralization in the metastatic bone microenvironment [21] and WB-MRI measures changes in the diffusivity of water molecules within tumors [20]. There is no good comparative evidence of significant superiority of any of these methods in measuring treatment response and no clear guidance on the preferred imaging technique in clinical practice [6].

Our hypothesis was that the three functional imaging methods, WB-MRI, FDG, and NaF PET/CT, can detect functional and metabolic changes in breast cancer skeletal metastases as early as 8 weeks after commencing endocrine-based therapy. The aims were to measure baseline parameters and endocrine therapy related changes with each method, to determine the accuracy of each method to predict progressive disease (PD), or non-PD compared to a clinical reference standard, and to determine if the magnitude of change in any method after 8 weeks of treatment was associated with progression-free survival (PFS).

## Patients and methods

This prospective study received research ethics committee approval and all patients gave signed informed consent. Thirty-one patients over the age of 18 with histologically confirmed breast cancer, with either de-novo or progressive bone-predominant metastatic disease scheduled for new endocrine therapy, were recruited. Patients who were also scheduled for radiotherapy or colony-stimulating factors were excluded due to potential effects on functional imaging parameters.

All patients underwent standard follow-up with clinical assessments including a pain inventory [22], blood tests, including serum alkaline phosphatase and tumor marker CA15–3, and standard imaging, including bone scintigraphy and/or diagnostic CT. The reference standard for clinical PD or non-PD was determined by two oncologists (IS and JM, with 10 and 27 years of specialist oncology experience) in consensus using all the listed clinical assessments up to 24 weeks, or earlier if there was clinical PD. Patients were categorized as either having clinical PD or non-PD (stable disease or partial response) as this is the most relevant dichotomization for clinical management, i.e., continue treatment if non-PD without treatment toxicity or change treatment if PD [23].

Prior to commencing endocrine therapy, patients underwent baseline WB-MRI, FDG, and NaF PET/CT, which were repeated using the same imaging protocol after 8 weeks of therapy. When an increase in maximum standardized uptake value (SUV_max_) was measured in any bone lesions, a further 12-week NaF PET/CT scan was performed when possible to help determine if an early increase in activity was due to the flare phenomenon, which has been reported with this tracer [24]. As a flare is not a recognized phenomenon with WB-MRI or FDG PET/CT, this was not performed with these modalities. As RECIST 1.1 precludes using CT for measuring response in bone metastases unless there is a measurable soft tissue component, we did not include stand-alone CT analysis in our protocol.

### WB-MRI

T1-weighted (T1-W), T2-weighted (T2-W), and diffusion-weighted (DWI, b-values: 50, 900 s/mm^2^) axial sequences were acquired for multiple bed positions from the base of the skull to upper thighs on a 1.5-T MRI scanner (Siemens Aera, Erlangen, Germany). Reformatted axial T1-W, T2-W, DWI b900, and apparent diffusion coefficient (ADC) map images (5-mm slice thickness and 1.2-mm in-plane pixel size) were produced for viewing, lesion identification, and analysis.

### FDG PET/CT

Scans were acquired 60 min after intravenous injection of FDG (mean 348 ± 18 MBq) and all patients had blood glucose measurements of < 10 mmol/l. Images were acquired from skull base to upper thighs with 3 min per bed position using a GE Discovery 710 PET/CT scanner (GE Healthcare, Chicago, IL, USA). A low-dose CT scan (140 kV, 10 mA, 0.5 s rotation time, and 40-mm collimation) was performed at the start of imaging to provide attenuation correction and an anatomical reference. PET images were reconstructed with a time-of-flight ordered subset expectation maximization algorithm (2 iterations, 24 subsets) with a reconstructed slice thickness of 3.27 mm and pixel size 4.7 mm.

### NaF PET/CT

Scans were acquired 60 min after injection of NaF (mean 228 ± 15 MBq). All other acquisition and reconstruction parameters were as for FDG PET/CT.

### Scan analysis

Up to five of the largest bone metastases as assessed on the NaF PET scans were analyzed in each patient by the same reader (GC), with 25 years of radiology and PET experience, using the identical lesions in each of the three scan types. For WB-MRI, lesions were identified on T1-W and T2-W sequences and regions drawn on DWI b900, which were automatically mapped to the accompanying ADC images for measurement of the median ADC in mm^2^/s (ADC_med_). A reduction in ADC_med_ of > 25% was used to differentiate imaging PD from imaging non-PD [25, 26]. For FDG and NaF PET/CT, the same lesions were selected and regions of interest (ROIs) outlined semi-automatically using a 40% of maximum activity threshold. The maximum standardized uptake value (SUV_max_) was measured from these regions and a > 25% increase used to differentiate imaging PD from imaging non-PD [27–29]. Individual lesion ADC_med_ and SUV_max_ measurements were recorded for per-lesion analysis and mean values for each patient recorded for per-patient analysis. Both analyses were performed to obtain clinically relevant results on a per-patient basis that can be used in management decisions and also to report on intra-patient response heterogeneity, a topic of interest in oncology and an observation that may impact on treatment decisions. Intra-patient inter-lesional heterogeneity of response was defined when a lesion showed a > 25% change that was discordant with the clinical reference standard for that patient. In NaF PET/CT scans, a flare was defined in each lesion that showed an initial increase in SUV_max_ at 8 weeks that then declined on the 12-week scan.

### Statistical methods

It was calculated that 20 patients with baseline and 8-week scans would give 80% power to predict clinical PD from non-PD 80% of the time (deemed a clinically useful level) for each modality. Differences between parameters in patients with clinical PD (PFS < 24 weeks) versus non-PD were tested for normality and compared using Student’s *t* test or Mann–Whitney *U* test as appropriate. PFS was defined as the time from the first study scan until the time to clinical progression. Patients who had not progressed clinically at the end of the study were censored. Relationships between scans and PFS were tested by comparing scan metrics in patients with PFS < 24 weeks with those > 24 weeks. Statistical analyses were conducted using IBM SPSS statistics software (version 24). A *P* value of < 0.05 was used for statistical significance.

## Results

### Patients

Twenty-two patients (median age 58.6, range, 40–79 years) completed at least one set of both baseline and 8-week imaging (18 all three modalities, two FDG alone, one FDG and NaF and one NaF alone) and hence WB-MRI: 18 patients, 76 lesions; FDG PET/CT: 21 patients, 90 lesions; NaF PET/CT: 20 patients, 85 lesions. Nine patients did not undergo 8-week imaging (eight due to patient choice and one required radiotherapy for incipient cord compression). Six patients had de novo metastatic disease and 16 had progressive disease prior to recruitment and apart from two patients who had small volume lung and liver metastases; all patients had only skeletal metastases. Endocrine therapy consisted of letrozole (*n* = 12), exemestane with everolimus (*n* = 6), tamoxifen (*n* = 3), and famotidine (*n* = 1). Bisphosphonates (zoledronic acid, *n* = 11 or ibandronate, *n* = 4) or denosumab (*n* = 7), were used as adjunctive therapy. By the clinical reference standard up to 24 weeks, five patients had PD and 17 patients had non-PD. Median PFS was 10.3 months (2.6–47.5 months) with 15 patients alive at the end of the study when censored.

### NaF PET/CT

**Fig. 1:**
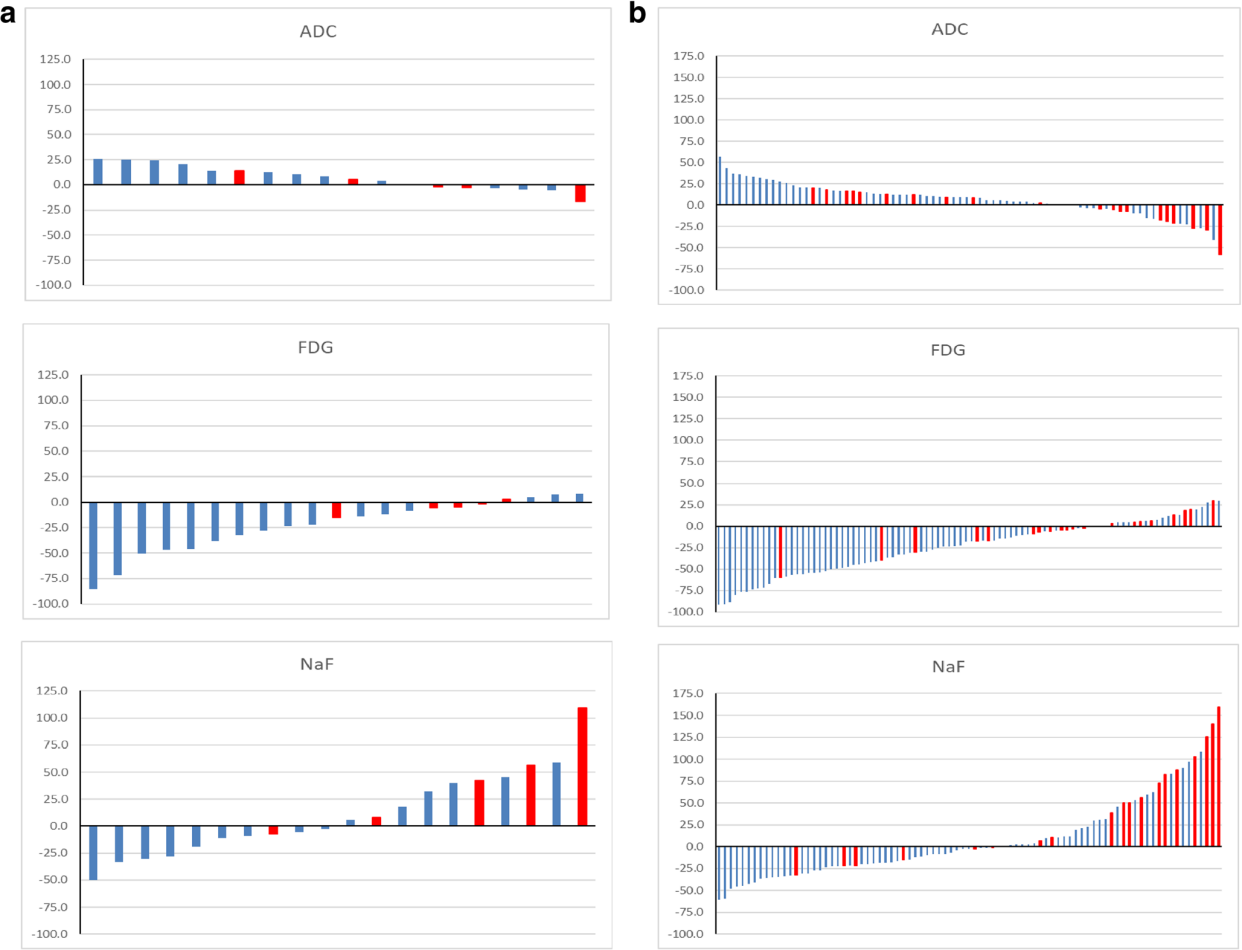
Waterfall plots showing % change in ADC_med_ and SUV_max_ in **a** each patient and in **b** each lesion for (*top*) WB-MRI, (*middle*) FDG PET/CT, and (*bottom*) NaF PET/CT [clinical non-progressors (*blue*) and progressors (*red*)]. ADC, apparent diffusion coefficient; FDG, [^18^F]-fluorodeoxyglucose; NaF, [^18^F]-sodium fluoride

There was a significant difference in % change in SUV_max_ between patients with clinical PD (PFS < 24 weeks) and non-PD on a per-patient analysis (41.7 vs. 0.7%, *p* = 0.039) and on a per-lesion analysis (44.4 vs. − 2.6%, *p* = 0.001). Of the 20 patients, 11 showed a less than 25% increase in SUV_max_ and were concordant with the clinical reference standard of non-PD. Three out of the five patients with clinical PD showed > 25% increase in SUV_max_ (Table 1, Fig. 1a). On analysis of the 85 lesions, 54 were concordant with the clinical reference standard of non-PD and 11 of 20 lesions showed a > 25% increase in SUV_max_ in patients with clinical PD (Table 1, Fig. 1b). Baseline SUV_max_ was not associated with clinical PD (*p* = 0.6).

**Table 1:** Performance of % changes in WB-MRI, FDG PET/CT and NaF PET/CT parameters in predicting clinical progressive disease up to 24 weeks on a per-patient and per-lesion basis

	Sensitivity (%)	Specificity (%)	PPV (%)	NPV (%)	Accuracy (%)
WB-MRI per patient	0	100	0	72.2	72.2
WB-MRI per lesion	15	96.4	60	76.1	75
FDG PET/CT per patient	0	100	0	76.2	76.2
FDG PET/CT per lesion	5	97.1	33.3	78.2	76.7
NaF PET/CT per patient	60	73.3	42.9	84.6	70.0
NaF PET/CT per lesion	55	83.1	50	85.7	76.5

*WB-MRI* whole-body magnetic resonance with diffusion-weighted imaging, *FDG* [^18^F]-fluorodeoxyglucose, *NaF* [^18^F]-sodium fluoride, *PET/CT* positron emission tomography/computed tomography

Twelve of the 85 lesions (14.1%) in seven patients showed discordant changes to the clinical reference standard and were categorized as showing an inter-lesional heterogeneous imaging response.

Eighteen lesions (21.2%) in seven patients showed an increase in NaF SUV_max_ at 8 weeks followed by a subsequent decrease at 12 weeks, and were therefore categorized as a flare. Four of these patients had clinical non-PD at 24 weeks.

### FDG PET/CT

There was a significant difference in % change in SUV_max_ between patients with clinical PD (PFS < 24 weeks) and non-PD on a per-patient analysis (− 4.8 vs. – 28.6%, *p* = 0.005) and on a per-lesion analysis (− 5.0 vs. − 29.7%, *p* = 0.001). Of the 21 patients, 16 showed < 25% increase in SUV_max_ and were concordant with the clinical reference standard of non-PD. None of the five patients with clinical PD showed an increase in SUV_max_ > 25% (Table 1, Fig. 1a). On analysis of the 90 individual lesions, 68 were concordant with clinical non-PD but only one of 20 lesions showed a > 25% increase in SUV_max_ in patients with clinical PD (Table 1, Fig. 1b). Baseline SUV_max_ was not associated with clinical PD (*p* = 0.65).

Five of the 90 lesions (5.6%) in four patients showed discordant changes to the clinical reference standard and were categorized as showing an inter-lesional heterogeneous imaging response.

### WB-MRI

There was no significant difference in % change in ADC_med_ between patients with clinical PD (PFS < 24 weeks) and non-PD on a per-patient analysis (− 0.5 vs. 10.1%, *p* = 0.098) but there was on a per-lesion analysis (− 3.2 vs. 9.2%, *p* = 0.012). Of the 18 patients, 13 showed less than a 25% decrease in ADC_med_ and were concordant with the clinical reference standard of non-PD. None of the five patients with clinical PD showed a > 25% decrease in ADC_med_ (Table 1, Fig. 1a). On analysis of the 76 individual lesions, 54 were concordant with clinical non-PD but only three of 20 lesions in patients with clinical PD showed a > 25% decrease in ADC_med_ (Table 1, Fig. 1b). There was no difference in baseline ADC_med_ (*p* = 0.46) between patients with PFS < 24 weeks compared to PFS > 24 weeks.

**Fig. 2:**
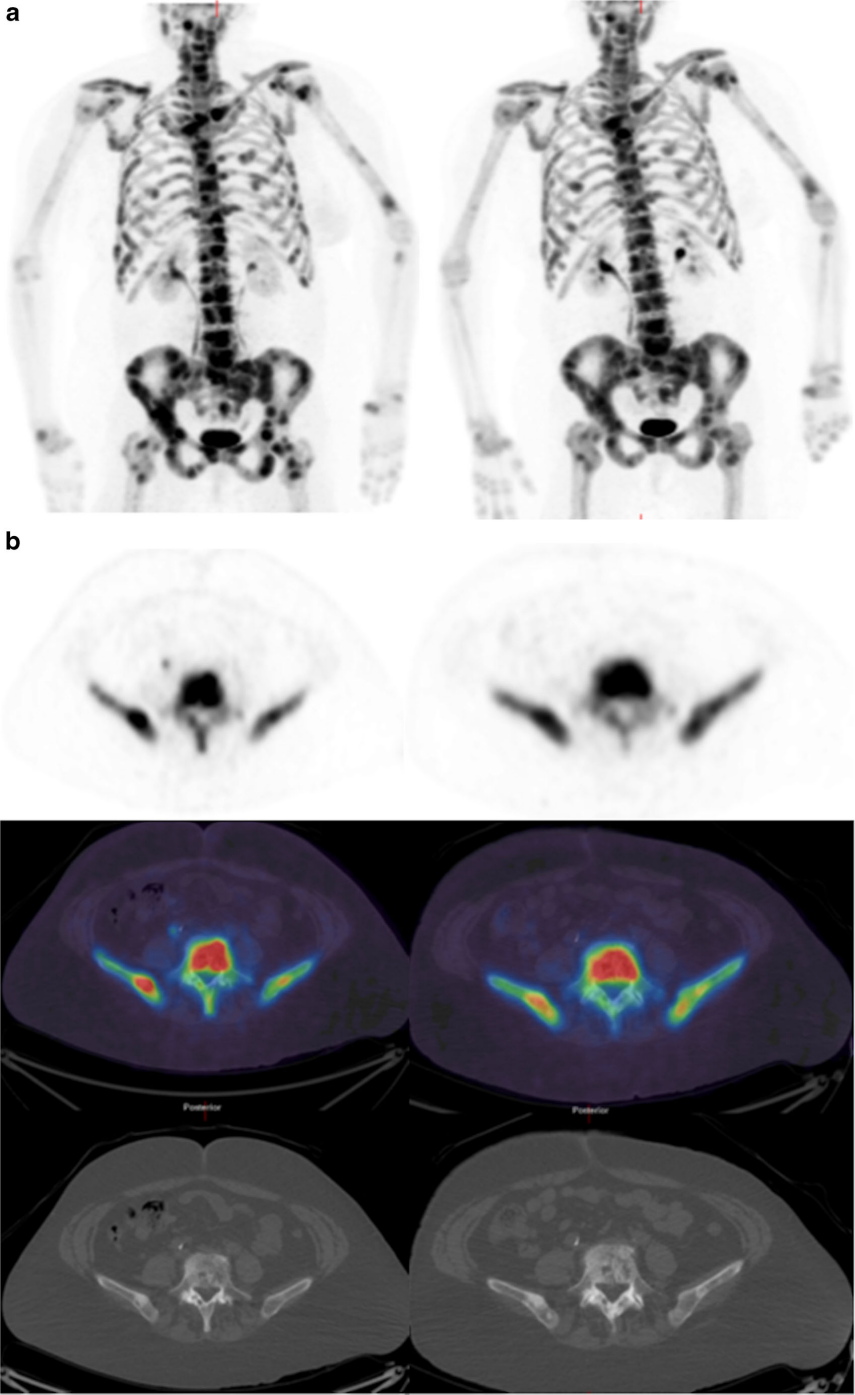
**a** NaF PET maximum intensity projection (MIP) and **b** corresponding transaxial images [PET (*top*), fused PET/CT (*middle*), and CT (*bottom*)] of a lesion in L5 at baseline (*left*) and 8 weeks (*right*) after commencing endocrine therapy in a patient who had a response by the clinical reference standard. SUV_max_ of the L5 vertebral lesion decreased from 42.7 to 32.5

**Fig. 3:**
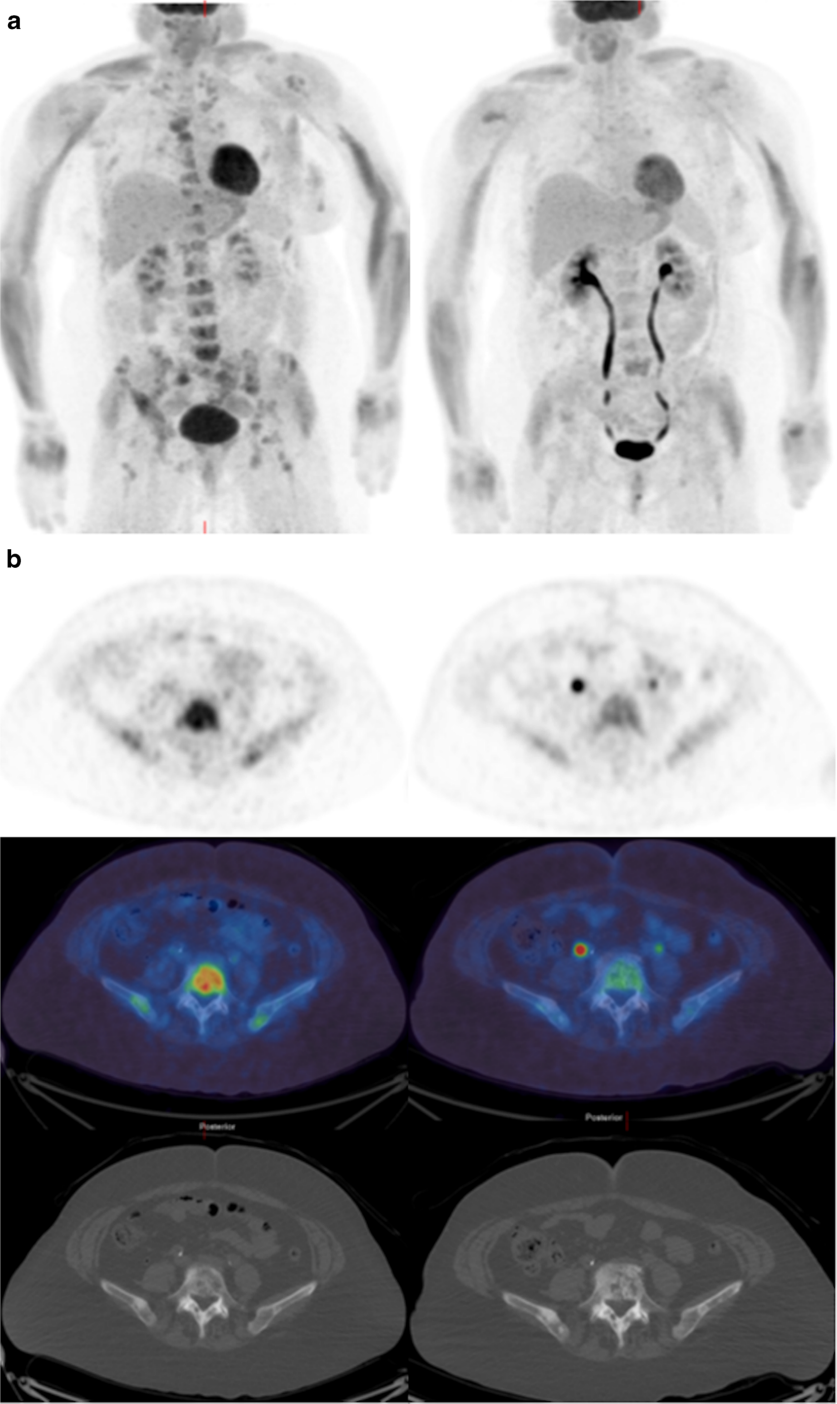
**a** FDG PET MIP and **b** corresponding transaxial images [PET (*top*), fused PET/CT (*middle*), and CT (*bottom*)] of a lesion in L5 at baseline (*left*) and 8 weeks (*right*) in the same patient as Figs. 2 and 4. SUV_max_ of the L5 vertebral lesion decreased from 9.9 to 4.9

**Fig. 4:**
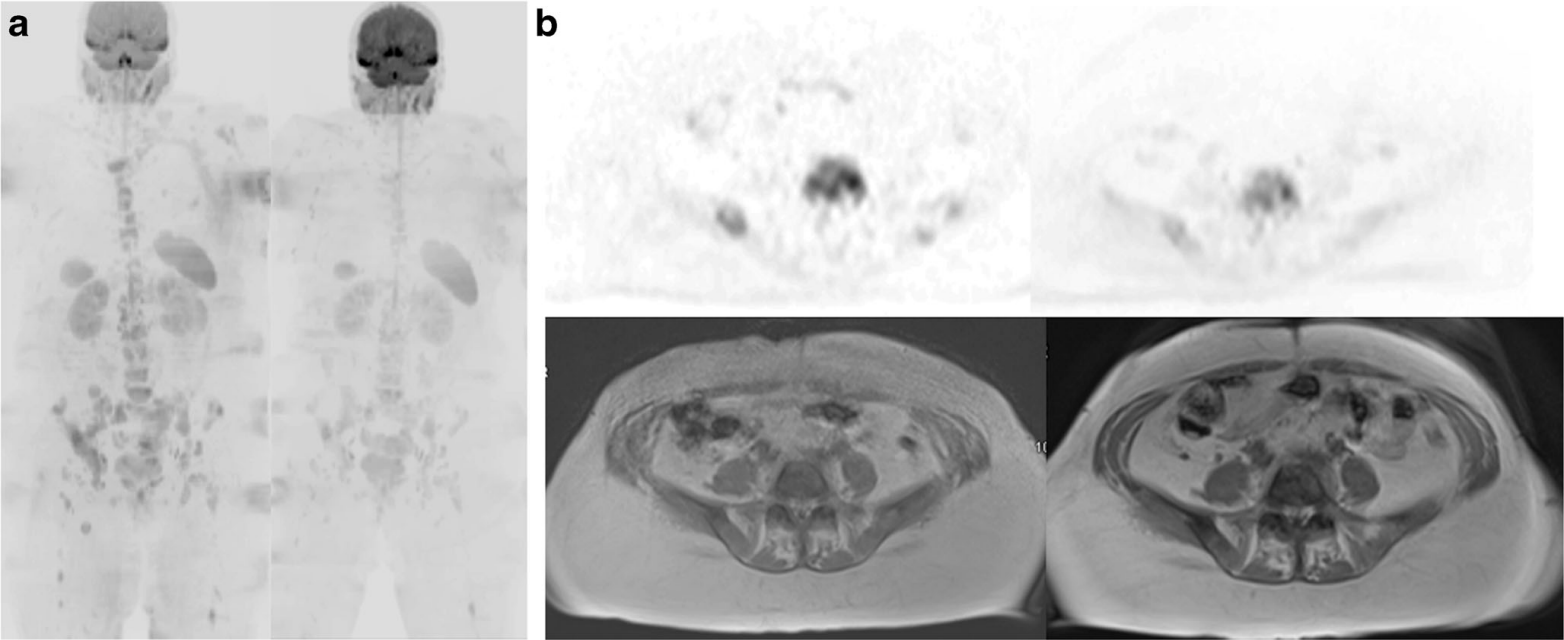
**a** b900 DWI MIP and **b** corresponding transaxial b900 DWI (*top*) and T1-W (*bottom*) images at baseline (*left*) and 8 weeks (*right*) of a L5 lesion in the same patient as Figs. 2 and 3. ADC_med_ of the L5 vertebral lesion increased from 1047 to 1150 mm^2^/s

Two of the 76 lesions (2.6%) in two patients showed discordant changes to the clinical reference standard and were categorized as showing an inter-lesional heterogeneous imaging response. Representative NaF, FDG PET, and WB-MRI images from a patient who showed a response by the clinical reference standard are illustrated in Figs. 2, 3, and 4, respectively.

## Discussion

Recognizing the limitations of conventional imaging in predicting treatment response in skeletal metastases and the increasing adoption of novel functional imaging into oncologic practice, it is timely to directly compare three contending functional imaging methods in this role. We have shown in this cohort of bone-predominant metastatic breast cancer patients treated with endocrine therapy that changes in parameters that reflect tumor cellularity (DWI), tumor glucose metabolism (FDG PET/CT), and the bone microenvironment (NaF PET/CT), can be detected and quantified. All three modalities showed a similar overall accuracy in predicting PD/non-PD as determined by a clinical reference standard that used conventional clinical, blood, and imaging methods up to 24 weeks. In addition, significant differences were seen in the magnitude of parameter change in those with PFS < 24 weeks compared to those with longer PFS, for NaF, and FDG PET/CT.

While FDG PET/CT and WB-MRI performed well in predicting non-PD, which would allow patients to continue with therapy [26], the magnitude of change (reduction in SUV_max_ or increase in ADC_med_) on a per-patient or per-lesion basis was greater with FDG (Fig. 1). However, neither WB-MRI nor FDG PET/CT predicted PD at this early 8-week time point. Both FDG PET/CT and WB-MRI primarily reflect tumor cell effects (glucose metabolism [30] and restriction in water molecule motion influenced by cellularity and other tumor-related factors [31], respectively) and while these demonstrated > 25% changes in less than 8 weeks in many responding metastases, the biological changes associated with tumor progression were not of sufficient magnitude to be detected this early at this threshold, implying a non-linear relationship between changes in image parameters and clinical PD.

Nevertheless, a significant difference in % change of FDG SUV_max_ was seen between patients with PFS < 24 weeks and those with longer PFS but with no significant difference in ADC_med_, implying % change in FDG SUV_max_ may be a better prognostic metric. Our findings augment previous reports where FDG PET/CT SUV_max_ has previously been shown to be associated with PFS in skeletal, nodal, or visceral metastases from breast cancer [8, 9] or to be associated with changes in tumor markers [10, 11]. To our knowledge, no literature exists for response prediction or assessment using WB-MRI in skeletal metastases from breast cancer in humans. Preclinical data, using a breast cancer model treated with the antiangiogenic agent bevacizumab, rather than endocrine treatment or chemotherapy, found DWI to be insensitive [32]. However, several small series report an increase in ADC in responding prostate cancer bone metastases [19, 20, 33].

While NaF PET/CT feasibility has previously been shown for monitoring treatment response in breast cancer bone metastases [13], to our knowledge definitive results have only been shown in prostate cancer [14–17]. In our series, NaF PET/CT showed modest sensitivity for predicting clinical PD (three of five patients). However, the clinical utility of NaF would be limited, as imaging PD would not be able to be differentiated from a treatment-induced flare, as observed in some of our patients. Despite these observations, the results for NaF PET/CT are of academic interest and suggest that the bone microenvironment changes reflected by this tracer are more rapid and larger in amplitude than the changes we saw with tumor cellular processes demonstrated by FDG PET/CT and WB-MRI.

We observed a heterogeneous response between metastases most frequently with NaF PET/CT, predominantly reflecting the flare phenomenon seen with this tracer. Some inter-lesional response heterogeneity was also observed with FDG PET/CT (5.6%) and WB-MRI (2.6%), suggesting that biological response heterogeneity exists and may reflect tumor resistance to therapy in some clones.

This study has some potential limitations. Additional test–retest scans were not performed for measurement of repeatability as the imaging protocol was already intensive for patients and as good repeatability and inter-observer variation have previously been reported for all three imaging methods employed in this study [19, 25, 26, 28, 29, 34]. Partly due to the intensity of the protocol, nine patients did not complete any 8-week imaging and a small number of patients did not undergo all three imaging tests. This led to a lower number of evaluable patients than preferred but nevertheless, enabled a comparison between all three modalities in most patients and a large number of metastases (*n* = 90) were included in the analysis. We adopted previously published thresholds of 25% change in image metrics to differentiate PD from non-PD but using alternative thresholds would not have significantly improved the ability to differentiate in this series with 25% appearing to be a satisfactory level cross the three modalities. Potential limitations with the clinical reference standard we employed were offset by using consensus from two blinded oncologists and allowing all standard clinical, blood, and imaging to be included while allowing up to 24 weeks for assessment in a method we have previously shown to be robust [35]. While specific treatments differed slightly between patients, they were all endocrine-based regimens without chemotherapy or other non-endocrine treatment-based regimes in order to minimize any heterogeneity due to different classes of treatment as was practically possible. Finally, we have only tested the imaging metrics in breast cancer patients undergoing endocrine-based therapy and we cannot exclude different biological effects from other therapeutic regimes, e.g., chemotherapy, that could affect imaging parameters differently.

## Conclusions

Changes in tumor cell characteristics and bone microenvironment can be measured with functional imaging methods in bone-predominant metastatic breast cancer at 8 weeks after commencing endocrine-based therapy, although the amplitude of changes did not always reach the threshold for response categorization. Overall accuracy in predicting PD is similar between the three tested modalities but FDG PET/CT and WB-MRI are more reliable than NaF PET/CT in determining non-PD at 8 weeks. Given the larger quantitative percent changes in FDG SUV_max_ compared to ADC_med_ and the fact that larger percent changes in FDG SUV_max_ are associated with PFS, this method has an advantage for determining non-PD and would allow an early decision for patient therapy to continue if there were no limiting side effects. In contrast, none of the three methods were reliable at 8 weeks in predicting subsequent clinical PD, NaF PET/CT performing best with a sensitivity of 60%. However, a flare and inter-lesional heterogeneity is relatively common at 8 weeks with NaF PET/CT and because of these factors, this method would not be sufficiently reliable to change a patient’s treatment at 8 weeks.
